# Evaluating the Measurement Properties of the Self-Assessment of Treatment Version II, Follow-Up Version, in Patients with Painful Diabetic Peripheral Neuropathy 

**DOI:** 10.1155/2017/6080648

**Published:** 2017-01-16

**Authors:** Floortje van Nooten, Dylan Trundell, Dorota Staniewska, Jun Chen, Evan W. Davies, Dennis A. Revicki

**Affiliations:** ^1^Astellas Pharma Global Development, Leiden, Netherlands; ^2^Evidera, London, UK; ^3^Evidera, Bethesda, MD, USA

## Abstract

*Background. *The Self-Assessment of Treatment version II (SAT II) measures treatment-related improvements in pain and impacts and impressions of treatment in neuropathic pain patients. The measure has baseline and follow-up versions. This study assesses the measurement properties of the SAT II.* Methods. *Data from 369 painful diabetic peripheral neuropathy (PDPN) patients from a phase III trial assessing capsaicin 8% patch (Qutenza®) efficacy and safety were used in these analyses. Reliability, convergent validity, known-groups validity, and responsiveness (using the Brief Pain Inventory-Diabetic Neuropathy [BPI-DN] and Patient Global Impression of Change [PGIC]) analyses were conducted, and minimally important differences (MID) were estimated.* Results. *Exploratory factor analysis supported a one-factor solution for the six impact items. The SAT II has good internal consistency (Cronbach's alpha: 0.96) and test-retest reliability (intraclass correlation coefficients: 0.62–0.88). Assessment of convergent validity showed moderate to strong correlations with change in other study endpoints. Scores varied significantly by level of pain intensity and sleep interference (*p* < 0.05) defined by the BPI-DN. Responsiveness was shown based on the PGIC. MID estimates ranged from 1.2 to 2.4 (pain improvement) and 1.0 to 2.0 (impact scores).* Conclusions. *The SAT II is a reliable and valid measure for assessing treatment improvement in PDPN patients.

## 1. Introduction

Neuropathic pain (NP) is a disorder of the central and peripheral nervous system resulting from a lesion or disease [1–3]. NP is one of the most prevalent pain aetiologies [3], with reported rates ranging from 0.9% to 8% of the general population [4, 5]. In diabetic patients, NP (referred to as diabetic polyneuropathy [DPN]) is one of the most common complications [3]. Painful diabetic peripheral neuropathy (PDPN) is a common form of DPN, with a prevalence of 5.8–34% in type I, type 2, or overall diabetes mellitus patients and an incidence of approximately 0.7 per 1000 persons per year [6]. In these patients, the system that signals pain is damaged or dysfunctional, resulting in symptoms such as aching, burning, shooting, and/or stabbing pain, often manifesting at night [3, 7]. Limbs and extremities are often affected, which subsequently impacts activities of daily living, sleep, work, and overall quality of life (QoL) [8].

The Initiative on Methods, Measurement, and Pain Assessment in Clinical Trials (IMMPACT) identified six core outcome domains as key for the assessment of efficacy and effectiveness of pain treatments: (1) pain; (2) physical functioning; (3) emotional wellbeing; (4) participant ratings of improvement and satisfaction with treatment; (5) symptoms and adverse events; and (6) participant disposition [9]. Patient-reported outcome (PRO) measures of pain, often used as primary endpoints, capture changes in pain intensity or frequency resulting from treatment but typically do not assess patient ratings of improvement and satisfaction. Furthermore, in a sample of patients with postoperative pain given the American Pain Society Satisfaction Survey, satisfaction was influenced by effectiveness of the medication independent of the level of pain intensity [10]. The five-item Self-Assessment of Treatment (SAT) questionnaire was developed based on the IMMPACT recommendations for assessing patient ratings of improvement and satisfaction [11, 12]. Items assess patient ratings of treatment benefit relating to pain, activity level, and QoL. Additionally, the SAT includes an item assessing if they would receive the treatment again and an item comparing treatments.

Despite strong evidence of the measurement properties of the SAT items [12], concerns were expressed about the lack of a recall period and about that the activity and QoL items covering too broad a construct for single items. Qualitative interviews were conducted with clinical experts and with patients diagnosed with NP [11]. Three clinicians provided their perspective on the most relevant symptoms and impacts of NP, as well as on the key benefits and harms associated with treatment. Additionally, the SAT was administered to 44 patients with NP, including PDPN (*N* = 20), human immunodeficiency virus-associated neuropathy (*N* = 16), and postherpetic neuropathy (*N* = 8), who provided feedback on both the measure and their experience with treatment for pain. The interviews confirmed the previous concerns with the activity and QoL items, with both being reported as too broad to capture key impacts. The activity item was subsequently split into three items, measuring improvements in self-care, daily activities, and physical activities. The QoL item was also split into three items, measuring improvements in sleep, emotional wellbeing, and social functioning. A recall period of 7 days was also added to the measure, and response options were adjusted for consistency between items. The modified measure, the SAT version II (SAT II), includes both baseline (measuring pain and impacts) and follow-up (measuring treatment-related improvements in pain and impacts and impressions of treatment) versions.

The SAT II was included in a phase III, double-blind, randomized, placebo-controlled clinical trial evaluating the efficacy and safety of capsaicin 8% patch (Qutenza) in subjects with PDPN [13]. The aim of this study was to develop the scoring algorithms for the SAT II follow-up version, including detecting and evaluating potential subscales, and to assess the measurement properties of these scores through psychometric evaluation.

## 2. Methods

### 2.1. Patient Sample

Data were collected from 369 patients with PDPN (≥3 Michigan Neuropathy Screening Instrument) from the phase III trial assessing the efficacy and safety of capsaicin 8% patch [13]. Patients included in the clinical trial had a score of ≥4 on the Brief Pain Inventory-Diabetic Neuropathy (BPI-DN) item 5 (a patient-reported measure of pain intensity evaluated on a 0–10 scale) at the screening visit and stable glycemic control when entering study. Patients had been diagnosed with painful, distal, symmetrical, sensorimotor polyneuropathy due to diabetes for at least 1 year prior to screening. They had at least one medical record of glycosylated hemoglobin (HbA1c) of <11.0% at 3–6 months before the screening visit and at screening, with variations of <1.0% between the 3- and 6-month prescreening value and screening value.

### 2.2. Study Design

The phase III trial was a double-blind, randomized, placebo-controlled, efficacy and safety study [13]. Subjects were randomly assigned to receive either a single application of capsaicin 8% patch or a placebo patch for 30 minutes at the baseline visit (day 1). This was followed by an observation period of 12 weeks involving four visits at weeks 2, 4, 8, and 12. The primary efficacy endpoint was the percent change in the BPI-DN item 5 score from baseline (average of daily scores during the week ending on day 1) to weeks 2–8 (average of daily scores during this period) in the active arm compared to the placebo arm. Data used to characterize the sample, including sociodemographic and clinical data, were collected at baseline or during screening.

### 2.3. PRO Measures

The SAT II follow-up version contains nine items in total. It measures the extent to which the study treatment has improved pain (question 1) and has six impact items assessing key impacts on QoL (self-care activities, daily activities, and physical activities [questions 2a–c]; emotional wellbeing, sleep, and social functioning [questions 3a–c]), all assessed on a five-point Likert scale using a 7-day recall period. Additionally, the extent to which a patient would be willing to receive the study treatment again (question 4) and impressions on how it compares to other treatments (question 5) are both captured using five-point Likert scales with no recall period stated. The SAT II follow-up version was administered at weeks 8 and 12.

The BPI-DN was developed to assess pain resulting from diabetes [14]. Item 5 of this measure assesses pain due to diabetes during the past 24 hours using an 11-point numerical rating scale (NRS; where 0 represents “no pain” and 10 represents “worst possible pain”). Item 9F assesses how pain interferes with sleep during the past 24 hours using an 11-point NRS (where 0 represents “does not interfere” and 10 represents “completely interferes”). A 30% reduction in pain severity using an 11-point NRS has previously been identified as a clinically important difference [15]. The BPI-DN items 5 and 9F were completed daily from first screening visit to week 12/end of study. For the 7-day average of daily BPI-DN item 5 and item 9F scores, data were considered nonmissing if scores were available from at least 4 days in the week. A 7-day average was calculated using 7 consecutive days ending on the day of the baseline, week 8 and week 12 visits.

The Patient Global Impression of Change (PGIC) measures the change in patients reported overall health status on a seven-point scale ranging from 1 (very much improved) to 7 (very much worse). In the phase III clinical trial, the PGIC was administered at weeks 2, 8, and 12.

The EuroQol-5 dimensions (EQ-5D) is a PRO measure developed to derive health utilities and is typically used in cost-utility analyses. The EQ-5D contains five items (pain/discomfort, self-care, mobility, anxiety/depression, and usual activities) that are scored using three-point Likert-type response scales. The responses are converted into a single index score using valuations of health states, based on the EQ-5D response options using the time trade-off method, in a representative sample of the general population [16]. The EQ-5D was completed at baseline and weeks 2, 8, and 12.

The Hospital Anxiety and Depression Scale (HADS) is a PRO measure developed to assess levels of anxiety and depression for use in clinical practice [17] but has also been used in numerous clinical trials. The HADS contains 14 items, with seven assessing depression and anxiety. Each item is scored using three-point Likert-type response scales. Summary scores for the anxiety and depression domains can be scored ranging from 0 to 21 with higher scores indicating greater anxiety/depression. The HADS was completed at baseline and weeks 2, 8, and 12.

The Neuropathic Pain Symptom Inventory (NPSI) is a PRO measure developed to evaluate different symptoms of neuropathic pain [18]. The NPSI contains 12 items, from which five summary pain scores can be calculated: burning, evoked, pressive, paroxysmal, and abnormal sensations. The 10 items used to derive the domain summary scores are each scored using a 0–10 NRS ranging from no pain/sensation to worst pain/sensation imaginable. The remaining two items report how consistently pain has been present and the number of pain episodes. The NPSI was completed at baseline and week 12.

### 2.4. Statistical Analyses

All subjects in the intent-to-treat-population with available PRO data, as required for each analysis, were included in the analysis sample, and no missing data were imputed. All statistical tests conducted were two-tailed with *p* < 0.05 used to determine significance. Due to similarities in the results for the follow-up version time points (weeks 8 and 12), unless specified otherwise, only week 12 data are reported.

#### 2.4.1. Patient Demographics and Clinical Characteristics

Demographic (gender, age, and race) and clinical variables (weight and concomitant medication use) collected at the baseline visit or during screening were used to characterize the patient sample.

#### 2.4.2. SAT II Descriptive Statistics

The distributional characteristics of the individual SAT II items were examined at week 8. Frequencies and percentages at each response level are reported to provide information on the range of response options used. In addition, the mean, standard deviation (SD), and median are reported for all items.

#### 2.4.3. Item-to-Item and Item-to-Scale Correlations

Spearman correlations were calculated to assess the relationship between the items (item-to-item correlations) and to provide information about the functioning of the instrument in the population. In addition, summary scores (combining items expected to be related) were correlated with the individual items (item-to-scale correlations). The analyses conducted at week 12 included correlations: between all SAT II items; between summary scores (the sum of the three activity items, the sum of the three QoL items, and the sum of all six impact items) and all items; and between the week average BPI-DN item 5 score and the pain item, the three summary scores, the treatment continuation item and treatment comparison item.

#### 2.4.4. Factor Analysis

To determine the number of domains and thus inform the scoring, exploratory factor analysis (EFA) was conducted at week 8 and at week 12. We included only the six impact items in the EFAs. The pain, treatment continuation, and treatment comparison items were not included, as they measure distinct and different concepts. Eigenvalues and the Root Mean Square Error of Approximation (RMSEA) were used to evaluate number of factors. Factor loadings >0.4 were considered acceptable (provided the loading is on one factor only).

#### 2.4.5. Scoring

The scoring approaches were based on the findings from the correlations and EFAs and derived after discussion among all authors. The pain, treatment continuation, and treatment comparison items were scored as individual items. The proposed scores were then assessed for reliability, validity, and ability to detect change.

#### 2.4.6. Reliability

The internal consistency reliability for the impact domain was assessed using Cronbach's formula for coefficient alpha at weeks 8 and 12. The target Cronbach's alpha is at least 0.70, though patterns of item-to-item correlations and item-to-total correlations are also important, as are the number of items in the subscale.

To measure test-retest reliability, stable patients were defined as those with a <20% change in BPI-DN item 5 (pain) score from week 8 to week 12 [15]. Stable patients were also defined using the definition of a change of <20% in EQ-5D Visual Analogue Scale (VAS) score. Intraclass correlation coefficients (ICCs) were calculated between week 8 and week 12 using SAT II follow-up scores. An ICC of >0.60 among stable subjects is considered acceptable to demonstrate test-retest reliability [19].

#### 2.4.7. Validity

Convergent validity was assessed via Spearman's rank-order correlation coefficient at weeks 8 and 12, between the SAT II scores and BPI-DN item 5 change from baseline score and change from previous week score (i.e., week 8 minus week 7; week 12 minus week 11); BPI-DN item 9F score; BPI-DN item 9F change from baseline score and change from previous week score; HADS subscale scores; HADS subscale change from baseline scores; PGIC; EQ-5D index and VAS scores; EQ-5D index and VAS change from baseline scores.

Known-groups validity for the follow-up version was examined at weeks 8 and 12 by analysis of variance (ANOVA) assessments comparing SAT II scores based on the following groups: BPI-DN item 5 score: 0–3, 4–6, and 7–10; and BPI-DN item 9F score: 0–3, 4–6, and 7–10. Pairwise comparisons between group means were assessed via *t*-tests. To account for multiple comparisons, Scheffé's method was applied.

#### 2.4.8. Responsiveness

An ANOVA was conducted at weeks 8 and 12 comparing responders and nonresponders defined using BPI-DN item 5 and the PGIC. Four separate analyses were conducted with responders defined as (1) BPI-DN item 5: ≥30% decline in pain severity; (2) BPI-DN item 5: ≥50% decline in pain severity; (3) PGIC: “minimally improved” or better; and (4) PGIC: “much improved” or better.

Nonresponders were defined as all patients not meeting those categories. Comparisons between responders and nonresponders were conducted for each of the SAT II scores.

#### 2.4.9. Minimally Important Scores

In the context of clinical trial use, while a measure may detect a difference between treatment arms, such an assessment does not consider whether or not the actual change experienced by patients is meaningful. A variety of methods have been developed to determine the minimum change in score that can be considered important, including both distribution- and anchor-based methods. Minimally important scores were estimated for the follow-up version SAT II scores.

One distribution-based approach which has been used for estimating minimally important scores is the standard error of measurement (SEM) [20, 21]. The SEM describes the error associated with the measure, in this case the SAT II scores, and is estimated by the SD of the measure multiplied by the square root of one minus its reliability coefficient (ICC from the test-retest assessment or Cronbach alpha from the internal consistency assessment). Shikiar et al. [22] found a general correspondence between the minimally important difference (MID) and SEM; however, this is somewhat dependent upon the magnitude of the reliability coefficient. SEM was calculated at week 8 and week 12. A second distribution-based approach conducted was an assessment of half of a SD of the SAT II scores at weeks 8 and 12. Norman et al. [23] suggest that one-half of a SD of a measure represents a clinically meaningful change, but not necessarily a MID. The half SD estimate provides an upper boundary for the MID. These analyses represent a statistical approach to defining minimally important scores and are considered supportive of anchor-based methods [24].

Anchor-based assessments select patients that achieve the MID for a measure that assesses a related construct (the anchor). The mean SAT II scores for this patient group represent minimally important scores, as it is assumed that patients that achieve a minimal response on the conceptually related anchor will also achieve a minimally important score on the SAT II. Minimally important scores were calculated at weeks 8 and 12, using the following anchors [15]: BPI-DN item 5 score change: 30%–40% and 25%–35%; BPI-DN item 9F score change: 30%–40% and 25%–35%; PGIC: minimally improved as well as minimally improved and much improved.

## 3. Results

### 3.1. Patient Demographics and Clinical Characteristics

The mean age at study baseline was 63.0 years, with a range of 33–89 years (Table 1). Patients were predominantly white (71.3%) and 58.3% were male. The mean weight was 93.4 kg and ranged from 46 to 151 kg. Concomitant medications were used by almost half of the sample (47.2%).

### 3.2. SAT II Descriptive Statistics

The SAT II follow-up descriptive characteristics were calculated at week 8 and week 12. At week 8, floor effects for the impact items 2a, 2b, 2c, 3a, 3b, and 3c (ranging from 39.8 to 50.9%) were identified. This is to be expected, given that not all patients are expected to improve and that the scale for these items does not include options that account for increased levels of pain (and subsequent impacts). Thirty-six percent (36%) of patients responded “yes, definitely” to a question if they would like to receive the treatment again, with a further 26% responding “yes, probably.” Fifty percent (50%) of patients report the treatment to be “somewhat better” or “very much better” than the other treatments they received for their condition. A very similar pattern of results was observed at week 12.

### 3.3. Item-to-Item and Item-to-Scale Correlations

Item-to-item correlations between the pain and impact items at week 12 ranged from 0.70 to 0.90. The correlation between the self-care item (2a) and daily activities item (2b) was particularly high (*r* = 0.90), indicating potential redundancy. However, given that the importance of both the self-care and daily activity items was established by patient interviews during the revision of the SAT and given the daily nature of self-care activities, both items seem to measure separate and important constructs. Correlations between the treatment comparison/treatment continuation items and the other items were typically lower, ranging from *r* = 0.50 to 0.69. Correlations between the pain and impact items with the activity summary, QoL summary, and impact summary scores were high (*r* = 0.78 to 0.95), indicating a strong relationship between these items.

### 3.4. Exploratory Factor Analysis

RMSEA was lower for a two-factor solution than a one-factor solution at week 8 (0.11 versus 0.19); however, correlations between the factors were relatively high (*r* = 0.68). Additionally, eigenvalues were dominated by a large first eigenvalue (4.8) with a value below 1.0 (0.62) for the second eigenvalue. Factor loadings were greater than 0.5 for all items in the one-factor solution at week 8 (ranging from 0.71 to 0.98). Combined, these findings support a one-factor solution for the six impact items. The findings were very similar between week 8 and week 12 factor analyses.

### 3.5. Scoring Approach

Factor analysis and item-to-scale correlations support a single factor for items 2a–c and 3a–c. These items should be scored as a single summary score using the mean of the constituent item scores. Using the mean allows for a more instinctive interpretation of the score back on the original five-point scale of the constituent items (i.e., ranging from “not at all” to “very much better”). Items 1, 4, and 5 should all be scored separately.

The following options are recommended for comparing treatment arms using the SAT II follow-up version: (1) compare mean item and summary scores by treatment arm, and/or (2) compare the proportions of patients by item response category or the proportion scorings above a specified threshold for each item.

### 3.6. Reliability

Internal consistency reliability, as assessed using Cronbach's alpha, was 0.86 for the impact domain at baseline, 0.96 at week 8, and 0.96 at week 12, indicating good internal consistency. Test-retest reliability was assessed among stable patients (with <20% change in BPI-DN item 5) at week 8 and week 12 (Table 2). Acceptable test-retest reliability was demonstrated for all of the follow-up scores (ICC range: 0.62–0.78). Among stable patients defined as <20% in EQ-5D VAS score, acceptable test-retest reliability was demonstrated for all of the follow-up scores (ICC range: 0.68–0.79; Table 2).

### 3.7. Validity

Convergent validity was assessed at week 8 and week 12, between the SAT II scores and the BPI-DN item 9F (sleep interference) score, the HADS subscale scores (anxiety and depression subscales), the EQ-5D index and VAS scores, and the NPSI domain scores (burning, evoked, pressive, paroxysmal, and abnormal sensations). Correlations ranged from 0.01 to −0.79 at week 8 and from −0.02 to −0.77 at week 12 and are presented in Tables 3(a) (week 8) and 3(b) (week 12).

At week 8, moderate to strong correlations were demonstrated on the BPI-DN item 5 and 9F overall and change from baseline scores for pain improvement, impact summary, treatment continuation, and treatment comparison (−0.30 to −0.60) (Table 3(a)). Strong correlations were demonstrated for all the items tested compared to the PGIC (−0.57 to −0.79), and moderate correlations were shown for the EQ-5D index overall and change from baseline (0.32 to 0.38). Weak correlations were shown between all SAT II items tested and HADS subscales and the EQ-5D VAS.

At week 12, moderate to strong correlations were also demonstrated on the BPI-DN item 5 and 9F overall and change from baseline scores for pain improvement, impact summary, treatment continuation, and treatment comparison (−0.29 to −0.58) (Table 3(b)). Strong correlations were demonstrated for all the items tested compared to the PGIC (−0.57 to −0.77). Moderate correlations were shown for the EQ-5D overall and change from baseline (0.32 to 0.38) and for the EQ-5D VAS (0.20 to 0.25). Weak correlations were shown between all SAT II items tested and HADS subscales.

For the known-groups validity analyses, *F* tests for ANOVAS were significant suggesting that the SAT II pain improvement, impact summary, treatment continuation, and treatment comparison scores discriminate between groups as defined by the BPI-DN item 5 (Table 4(a)) and item 9F (Table 4(b)) at weeks 8 and 12. Scheffé's post hoc tests demonstrated that all scores were significantly different between the 0–4 versus 4–6 and 0–4 versus 7–10 categories on the BPI-DN items 5 and 9F for weeks 8 and 12. Scores were also significantly different between the 4–6 and 7–10 categories for the pain improvement and treatment comparison scores at week 8, and for the pain improvement at week 12 on the BPI-DN item 5, as well as at week 8 on the pain improvement scores on the BPI-DN item 9F.

### 3.8. Responsiveness

At both weeks 8 and 12, significant differences (*p* < 0.0001) were demonstrated for all items between responders and nonresponders, based on a responder definition of a ≥ 30% and a ≥ 50% reduction in BPI-DN item 5 score. The general trend for all items is that there are a greater proportion of responders than nonresponders in categories indicating superior benefit (e.g., “quite a bit better” and “very much better”).

When using the PGIC to define responders, significant differences were observed between responders and nonresponders (*p* < 0.0001) for all items at both weeks 8 and 12, using both definitions (i.e., “minimally improved” or better or “much improved” or better, to define responders). The general trend for all items is that there is a greater proportion of responders than nonresponders in categories indicating superior benefit (e.g., “quite a bit better” and “very much better”). These results demonstrate that the SAT II items and summary scores were able to detect a clinically meaningful change in health status or level of pain.

### 3.9. Minimally Important Scores

Table 5 presents the percentage of patients meeting minimally important score estimates for the SAT II follow-up scores at week 12. The SEM was calculated at week 8 and week 12 using the ICC for both the BPI-DN item 5 and the EQ-5D VAS. When using the ICC based on the BPI-DN item 5, the SEM and (1/2)SD values were consistent across time points for pain improvement (SEM = 0.64 and 0.65; (1/2)SD = 0.68 and 0.69), impact summary (SEM = 0.62 and 0.63; (1/2)SD = 0.62 and 0.62), treatment continuation (SEM = 0.76 and 0.85; (1/2)SD = 0.62 and 0.69), and treatment comparison (SEM = 0.52 and 0.55; (1/2)SD = 0.46 and 0.49). When using the ICC based on the EQ-5D VAS, the SEM and (1/2)SD values were also consistent across time points for pain improvement (SEM = 0.71 and 0.72; (1/2)SD = 0.68 and 0.69), impact summary (SEM = 0.56 and 0.56; (1/2)SD = 0.62 and 0.62), treatment continuation (SEM = 0.66 and 0.73; (1/2)SD = 0.62 and 0.69), and treatment comparison (SEM = 0.52 and 0.55; (1/2)SD = 0.46 and 0.49).

Based on the total summary of evidence on estimates of minimally important scores, with greater focus on the anchor-based estimates, attainment of a pain improvement score of 1.2 to 2.4 may represent a meaningful threshold for determining clinically meaningful improvement. For impact summary scores, a score of 1.0 to 2.0 may represent a meaningful threshold for determining clinically meaningful improvement. Based on all the anchor-based estimates, a score of ≥1.5 may be considered clinically meaningful for both the pain improvement and impact summary scores; a more conservative estimate would be a score of ≥2.0. Treatment continuation and treatment comparison scores are directly translatable.

## 4. Discussion

The SAT II questionnaire is based on the original SAT questionnaire developed based on the IMMPACT recommendations. The original measure lacked a recall period and the activity and QoL items were considered too broad [12]. The need for a new version of the questionnaire was identified through qualitative research, which showed that the original questionnaire was lacking in content validity for the activity and QoL items. The modified SAT II measure was developed to address these concerns [11]. There are two versions of the SAT II, one to be administered at baseline and the other at follow-up visits. The SAT II baseline version contains seven items evaluating current status (pain level, impact on self-care activities, daily activities, physical activities, emotional wellbeing, and sleep and social functioning). The SAT II follow-up version contains nine items: seven items similar to the baseline version but asking about the improvement on the level or impact of pain due to treatment and two additional items on whether (1) the patient wants to receive the treatment again and (2) how the treatment compares with other pain treatments. The SAT II baseline version is recommended for use in characterizing the patient sample, while the SAT II follow-up version is recommended for use to compare patient-reported improvements by treatment arm. This study focused on the development of scoring algorithms and the assessment of the measurement properties of the SAT II follow-up version for use in clinical trials.

The analyses reported here support combining items 2a–c (daily and physical activities) and 3a–c (emotional wellbeing, sleep and social functioning) as a single summary score. Item-to-item correlations were generally moderate to strong. This relationship was further explored by factor analysis, which supported the use of a single summary score comprising items 2a–c and 3a–c. There are two acceptable approaches to scoring. The first is simply to compare mean scores by treatment arm for questions 1, 4, and 5 and the summary score (questions 2-3). The second method compares the proportions of patients reporting different SAT II items response levels by treatment arm. For this analysis, rather than comparing across all response categories, patients can be grouped as those at or above versus below a response category (e.g., “moderately better”) and compared by treatment arm (i.e., a 2 × 2 contingency table; and odds ratios and chi-square* p* values can be reported).

The SAT II demonstrated good internal consistency and good test-retest reliability (both the BPI-DN item 5 and the EQ-5D VAS were used to define stable patients). Tests of convergent validity showed that the BPI-DN items 5 and 9F and the PGIC were most strongly correlated with SAT II score at both weeks 8 and 12, while the EQ-5D showed moderate correlations. The weakest correlations were seen in relation to the EQ-5D VAS and the HADS. Tests for known-groups validity showed that the SAT II scores varied significantly by level of pain intensity and sleep interference. SAT II scores clearly delineated between pain severity and sleep interference groups, with better SAT II scores in the groups reporting lower pain severity or sleep interference.

The distribution-based minimally important scores were consistent across time points when using the ICC based on either the BPI-DN item 5 or the EQ-5D VAS. Based on the overall anchor-based estimates, a score of ≥1.5 at follow-up may be considered clinically meaningful for both the pain improvement and impact summary scores; a more conservative estimate would be a score of ≥2.0. Note that an achieved score of 2.0 is equivalent to “moderately better” or greater, and an achieved score of 1.5 is equivalent to the case between slightly and moderately better. Based on the results, a threshold of 2.0 may provide the best estimate for clinical significance. The treatment continuation and treatment comparison scores are directly translatable where a treatment continuation score of 3 or greater represents yes probably or yes definitely, and for treatment comparison a score of 3 or greater represents somewhat or very much better.

To be used as an endpoint in clinical trials, it is not enough for a measure to be reliable and valid but it needs to also be sensitive to changes in a patient's condition. The SAT II has been shown to be very responsive to change, based on a measure of patient-reported global change and to an improvement in pain severity scores. Responsiveness was demonstrated for all item scores across all responder definitions. In addition, the SAT II impact summary score (items 2-3) also demonstrated ability to detect change based on the patient global ratings and improvements in pain severity.

## 5. Conclusion

The SAT II follow-up version measures patient-reported improvement in pain and impact of treatment on daily activities and functioning and treatment satisfaction. The SAT II follow-up version demonstrated good internal consistency and test-retest reliability and good evidence supporting convergent and known group's validity. More importantly, the SAT II was responsive to changes in pain severity and global ratings of change in health status. The SAT II may be an acceptable endpoint for pain treatment studies. These findings suggest that the SAT II may be an acceptable primary or secondary endpoint in PDPN clinical trials. Future research is needed to confirm the measurement properties of the SAT II.

## Figures and Tables

**Table 1:** Demographic and clinical characteristics at baseline.

Characteristic	All patients (*N* = 369)
*Age*	
Mean (SD)	63.0 (10.8)
Range	33–89
*Gender (N, %)*	
Female	154 (41.7%)
Male	215 (58.3%)
*Race (N, %)*	
American Indian or Alaska Native	3 (0.8%)
Black or African-American	74 (20.1%)
Native Hawaiian or other Pacific Islander	3 (0.8%)
Asian	8 (2.2%)
White	263 (71.3%)
Other	18 (4.9%)
*Weight (kg)*	
Mean (SD)	93.4 (16.6)
Range	46–151
*Use of concomitant medication (N, %)*	
Yes	174 (47.2%)
No	195 (52.8%)

SD: standard deviation.

**Table 2:** Test-retest reliability of SAT II follow-up scores among stable subjects (EQ-5D VAS < 20% and BPI-DN item 5 < 20%): week 8 to week 12.

SAT II domains	EQ-5D VAS < 20%^1^					BPI-DN item 5 < 20%^2^				
	*N*	Mean (SD) week 8	Mean (SD) week 12	Difference^3^	ICC^4^	*N*	Mean (SD) week 8	Mean (SD) week 12	Difference	ICC
Pain improvement	253	1.34 (1.36)	1.18 (1.41)	−0.16	0.73	213	0.98 (1.20)	0.86 (1.22)	−0.11	0.78
Impact summary	247	1.14 (1.25)	1.07 (1.28)	−0.08	0.79	209	0.87 (1.06)	0.80 (1.09)	−0.07	0.75
Treatment continuation	246	2.78 (1.25)	2.65 (1.36)	−0.13	0.72	207	2.62 (1.25)	2.35 (1.40)	−0.27	0.62
Treatment comparison	244	2.69 (0.92)	2.58 (0.98)	−0.11	0.68	205	2.50 (0.85)	2.40 (0.95)	−0.09	0.68

^1^Stable defined as <20% change on EQ-5D VAS score from week 8 to week 12.

^2^Stable defined as <20% change on BPI-DN item 5 from week 8 to week 12.

^3^Difference = week 12 mean − week 8 mean.

^4^Intraclass correlation coefficient.

**(a):** 

Measures	SAT II scores			
	Pain improvement	Impact summary	Treatment continuation	Treatment comparison
BPI-DN item 5	−0.55^*∗∗∗*^	−0.46^*∗∗∗*^	−0.30^*∗∗∗*^	−0.51^*∗∗∗*^
BPI-DN item 5 change from baseline	−0.60^*∗∗∗*^	−0.54^*∗∗∗*^	−0.40^*∗∗∗*^	−0.60^*∗∗∗*^

BPI-DN item 9F	−0.42^*∗∗∗*^	−0.34^*∗∗∗*^	−0.22^*∗∗∗*^	−0.40^*∗∗∗*^
BPI-DN item 9F change from baseline	−0.55^*∗∗∗*^	−0.55^*∗∗∗*^	−0.35^*∗∗∗*^	−0.61^*∗∗∗*^

HADS anxiety subscale	−0.10	−0.07	−0.09	−0.12^*∗*^
HADS anxiety subscale change from baseline	−0.08	−0.09	−0.09	−0.10
HADS depression subscale	−0.11^*∗*^	−0.10	−0.10	−0.11^*∗*^
HADS depression subscale change from baseline	−0.13^*∗*^	−0.13^*∗*^	−0.14^*∗*^	−0.19^*∗∗*^
PGIC	−0.79^*∗∗∗*^	−0.76^*∗∗∗*^	−0.57^*∗∗∗*^	−0.78^*∗∗∗*^
EQ-5D index	0.30^*∗∗∗*^	0.26^*∗∗∗*^	0.22^*∗∗∗*^	0.30^*∗∗∗*^
EQ-5D index change from baseline	0.32^*∗∗∗*^	0.31^*∗∗∗*^	0.30^*∗∗∗*^	0.38^*∗∗∗*^
EQ-5D VAS	0.20^*∗∗*^	0.16^*∗*^	0.16^*∗*^	0.19^*∗∗*^
EQ-5D VAS change from baseline	0.19^*∗∗*^	0.19^*∗∗*^	0.15^*∗*^	0.17^*∗*^

^1^Spearman's rank order correlation. ^*∗*^*p* < 0.05; ^*∗∗*^*p* < 0.001; ^*∗∗∗*^*p* < 0.0001.

**(b):** 

Measures	SAT II scores			
	Pain improvement	Impact summary	Treatment continuation	Treatment comparison
BPI-DN item 5	−0.54^*∗∗∗*^	−0.42^*∗∗∗*^	−0.29^*∗∗∗*^	−0.39^*∗∗∗*^
BPI-DN item 5 change from baseline	−0.58^*∗∗∗*^	−0.52^*∗∗∗*^	−0.42^*∗∗∗*^	−0.47^*∗∗∗*^

BPI-DN item 9F	−0.42^*∗∗∗*^	−0.31^*∗∗∗*^	−0.21^*∗∗*^	−0.35^*∗∗∗*^
BPI-DN item 9F change from baseline	−0.53^*∗∗∗*^	−0.49^*∗∗∗*^	−0.36^*∗∗∗*^	−0.47^*∗∗∗*^

HADS anxiety subscale	−0.10	−0.06	−0.08	−0.12^*∗*^
HADS anxiety subscale change from baseline	−0.06	−0.10	−0.04	−0.06
HADS depression subscale	−0.09	−0.09	−0.09	−0.11
HADS depression subscale change from baseline	−0.12^*∗*^	−0.18^*∗∗*^	−0.13^*∗*^	−0.14^*∗*^
PGIC	−0.77^*∗∗∗*^	−0.74^*∗∗∗*^	−0.57^*∗∗∗*^	−0.74^*∗∗∗*^
EQ-5D index	0.30^*∗∗∗*^	0.28^*∗∗∗*^	0.18^*∗*^	0.23^*∗∗∗*^
EQ-5D index change from baseline	0.27^*∗∗∗*^	0.30^*∗∗∗*^	0.27^*∗∗∗*^	0.24^*∗∗∗*^
EQ-5D VAS	0.23^*∗∗∗*^	0.24^*∗∗∗*^	0.20^*∗∗*^	0.25^*∗∗∗*^
EQ-5D VAS change from baseline	0.20^*∗∗*^	0.23^*∗∗∗*^	0.21^*∗∗*^	0.22^*∗∗∗*^

^1^Spearman's rank order correlation. ^*∗*^*p* < 0.05; ^*∗∗*^*p* < 0.001; ^*∗∗∗*^*p* < 0.0001.

**(a):** 

SAT II follow-up scores	BPI-DN item 5			Overall *F* value^2^ (*p* value)	Pairwise comparisons^3^
	0–<4 *N*, mean (SD)^1^	4–6 *N*, mean (SD)^1^	>6–10 *N*, mean (SD)^1^		
Week 8					
Pain improvement	124, 2.15 (1.33)	100, 1.00 (1.20)	111, 0.57 (0.86)	59.14^*∗∗∗*^	1^*∗∗∗*^, 2^*∗∗∗*^, 3^*∗*^
Impact summary	123, 1.77 (1.35)	100, 0.92 (1.04)	109, 0.57 (0.83)	35.74^*∗∗∗*^	1^*∗∗∗*^, 2^*∗∗∗*^
Treatment continuation	122, 3.09 (1.13)	99, 2.63 (1.23)	109, 2.45 (1.26)	8.77^*∗∗*^	1^*∗*^, 2^*∗∗∗*^
Treatment comparison	122, 3.18 (0.90)	99, 2.57 (0.86)	109, 2.25 (0.75)	36.99^*∗∗∗*^	1^*∗∗∗*^, 2^*∗∗∗*^, 3^*∗*^
Week 12					
Pain improvement	116, 2.06 (1.49)	109, 0.87 (1.15)	92, 0.37 (0.67)	57.27^*∗∗∗*^	1^*∗∗∗*^, 2^*∗∗∗*^, 3^*∗*^
Impact summary	115, 1.64 (1.35)	108, 0.87 (1.11)	91, 0.54 (0.86)	26.05^*∗∗∗*^	1^*∗∗∗*^, 2^*∗∗∗*^
Treatment continuation	115, 3.17 (1.07)	108, 2.35 (1.39)	91, 2.22 (1.50)	16.33^*∗∗∗*^	1^*∗∗∗*^, 2^*∗∗∗*^
Treatment comparison	113, 2.97 (0.94)	108, 2.46 (0.96)	90, 2.21 (0.92)	17.62^*∗∗∗*^	1^*∗∗∗*^, 2^*∗∗∗*^

^1^SAT II mean scores and SD for patients with a BPI-DN item 5 score within the specified range.

^2^An analysis of variance (ANOVA).

^3^Pairwise comparisons between means were performed using Scheffé's test adjusting for multiple comparisons; 1 = 0–3 versus 4–6; 2 = 0–3 versus 7–10; 3 = 4–6 versus 7–10; ^*∗*^*p* < 0.05, ^*∗∗*^*p* < 0.001, and ^*∗∗∗*^*p* < 0.0001.

**(b):** 

SAT II follow-up scores	BPI-DN item 9F			Overall *F* value^2^ (*p*-value)	Pairwise comparisons^3^
	0–3 *N*, mean (SD)^1^	4–6 *N*, mean (SD)^1^	7–10 *N*, mean (SD)^1^		
Week 8					
Pain improvement	186, 1.73 (1.41)	86, 0.95 (1.14)	63, 0.41 (0.66)	30.80^*∗∗∗*^	1^*∗∗∗*^, 2^*∗∗∗*^, 3^*∗*^
Impact summary	184, 1.49 (1.34)	85, 0.81 (0.93)	63, 0.46 (0.71)	23.21^*∗∗∗*^	1^*∗∗∗*^, 2^*∗∗∗*^
Treatment continuation	182, 2.97 (1.17)	85, 2.45 (1.27)	63, 2.46 (1.24)	7.55^*∗∗*^	1^*∗∗*^, 2^*∗*^
Treatment comparison	182, 2.97 (0.92)	85, 2.49 (0.88)	63, 2.14 (0.69)	23.91^*∗∗∗*^	1^*∗∗∗*^, 2^*∗∗∗*^
Week 12					
Pain improvement	176, 1.66 (1.49)	79, 0.61 (1.01)	62, 0.45 (0.74)	31.00^*∗∗∗*^	1^*∗∗∗*^, 2^*∗∗∗*^
Impact summary	175, 1.37 (1.34)	77, 0.72 (1.01)	62, 0.58 (0.84)	14.55^*∗∗∗*^	1^*∗∗∗*^, 2^*∗∗∗*^
Treatment continuation	175, 2.85 (1.28)	77, 2.34 (1.39)	62, 2.27 (1.51)	6.23^*∗∗*^	1^*∗*^, 2^*∗*^
Treatment comparison	172, 2.84 (0.95)	77, 2.38 (0.93)	62, 2.08 (0.95)	17.20^*∗∗∗*^	1^*∗∗*^, 2^*∗∗∗*^

^1^SAT II mean scores and SD for patients with a BPI-DN item 9F score within the specified range.

^2^An analysis of variance (ANOVA).

^3^Pairwise comparisons between means were performed using Scheffé's test adjusting for multiple comparisons; 1 = 0–3 versus 4–6; 2 = 0–3 versus 7–10; 3 = 4–6 versus 7–10; ^*∗*^*p* < 0.05, ^*∗∗*^*p* < 0.001, and ^*∗∗∗*^*p* < 0.0001.

**Table 5:** Percentage of participants meeting various MID definitions for SAT II follow-up scores at week 12.

SAT II follow-up score	SEM^1^	SEM^2^	0.5 SD	BPI-DN item 5: 30–40%	BPI-DN item 5: 25–35%	BPI-DN item 9F: 30–40%	BPI-DN item 9F: 25–35%	PGIC: minimally improved	PGIC: minimally improved and much improved
Pain improvement	175 (51.62%)	175 (51.62%)	175 (51.62%)	108 (31.86%)	108 (31.86%)	108 (31.86%)	108 (31.86%)	74 (21.83%)	74 (21.83%)
Impact summary	166 (48.97%)	166 (48.97%)	166 (48.97%)	127 (37.46%)	117 (34.51%)	107 (31.56%)	127 (37.46%)	75 (22.12%)	64 (18.88%)
Treatment continuation	292 (86.14%)	292 (86.14%)	292 (86.14%)	123 (36.28%)	123 (36.28%)	123 (36.28%)	123 (36.28%)	123 (36.28%)	123 (36.28%)
Treatment comparison	317 (93.51%)	317 (93.51%)	317 (93.51%)	154 (45.43%)	154 (45.43%)	154 (45.43%)	154 (45.43%)	71 (20.94%)	71 (20.94%)

SEM = SD of the measure multiplied by the square root of 1 minus its reliability coefficient ICC from the test-retest assessment.

^1^ICC1: evaluated among stable subjects (BPI-DN item 5 < 20%) between week 8 and week 12.

^2^ICC2: evaluated among stable subjects (EQ-5D VAS change < 20%) between week 8 and week 12.
